# Uncovering Novel DPP-IV Inhibitory Peptides from Amphibian (*Lithobates catesbeiana*) Skin via Peptidomics and Molecular Simulation

**DOI:** 10.3390/foods14173023

**Published:** 2025-08-28

**Authors:** Zongmu Fang, Mei Zhang, Junhui Lian, Yangqing Xiao, Donghui Luo, Mouming Zhao, Lianzhu Lin

**Affiliations:** 1 Chaozhou Branch of Chemistry and Chemical Engineering Guangdong Laboratory, Chaozhou 521000, China; 2 School of Food Science and Engineering, South China University of Technology, Guangzhou 510640, China

**Keywords:** *Lithobates catesbeiana* skin gelatin, DPP-IV inhibition, peptide, *in silico* analysis

## Abstract

As an emerging natural source of DPP-IV inhibition strategy, we report for the first time the use of *Lithobates catesbeianus* skin gelatin (LSG) as a novel source for DPP-IV inhibitory peptides in this study. Through enzymatic hydrolysis with multiple proteases, the papain-treated hydrolysate exhibited superior performance in hydrolysis degree, protein recovery, and DPP-IV inhibition, with 93.47% of peptides under 1 kDa. Subsequent separation and peptidomics analysis identified 13 previously unreported peptides. Molecular docking and *in silico* screening pinpointed four candidate peptides, i.e., LGPQR, RGFDQ, RGPVGP, and RLDDVT, which were then synthesized and functionally validated. Enzyme kinetic studies revealed that these peptides acted via competitive or mixed-type inhibition mechanisms. Notably, this study uncovered the bio-functional potential of amphibian-derived gelatin and provided a new strategy for natural DPP-IV inhibitor discovery through integrated enzymatic, computational, and biochemical approaches. This work pioneered the use of amphibian skin gelatin in antidiabetic peptide discovery and laid the foundation for its application in functional foods.

## 1. Introduction

Diabetes mellitus ranks as the third most prevalent chronic metabolic disorder globally, following cardiovascular diseases and cancer. According to the 2021 report by the International Diabetes Federation (IDF), approximately 537 million adults were living with diabetes, and this number is projected to increase to 643 million by 2030. Diabetes mellitus and its complications account for nearly 6.7 million deaths annually, representing 12.2% of global mortality in the adult population (https://diabetesatlas.org, accessed on 20 January 2025). Diabetes mellitus is a metabolic disorder characterized by chronic hyperglycemia resulting from defects in insulin secretion, insulin action, or both, often accompanied by disturbances in carbohydrate, fat, and protein metabolism (https://www.who.int, accessed on 20 January 2025). It is commonly classified into four types: type 1 diabetes mellitus, type 2 diabetes mellitus (T2D), gestational diabetes mellitus, and other specific types. Among them, T2D accounts for approximately 90% of all diabetes mellitus cases (https://diabetesatlas.org, accessed on 21 January 2025) and is recognized as a complex, polygenic disease associated with insulin resistance and β-cell dysfunction, leading to impaired glucose homeostasis [1]. Consequently, therapeutic strategies aimed at enhancing insulin sensitivity and restoring glucose regulation are of paramount importance in managing T2D.

Dipeptidyl peptidase-IV (DPP-IV) is a serine protease broadly distributed in human tissues, including intestinal epithelium, kidney, and vascular endothelium [2]. Its primary substrates include incretin hormones such as glucagon-like peptide-1 (GLP-1) and gastric inhibitory polypeptide (GIP), which regulate blood glucose by modulating insulin and glucagon secretion via pancreatic α- and β-cell receptors [3]. In individuals with T2D, DPP-IV activity is significantly elevated, accelerating the degradation of GLP-1 and thereby reducing its glucose-lowering efficacy [4]. As such, DPP-IV inhibition has emerged as a validated therapeutic approach in T2D management.

Bioactive peptides, comprising short amino acid sequences with specific physiological functions, can be derived either endogenously or produced exogenously via enzymatic hydrolysis or biotechnological processing of food proteins [5,6]. Due to their low molecular weight and minimal immunogenicity, these peptides are readily absorbed and can cross physiological barriers, including the blood–brain barrier, to exert systemic effects [7,8]. Previous studies have reported DPP-IV inhibitory peptides with IC_50_ values ranging from 2.39 to 2.93 mg/mL derived from plant sources such as soybean, pea, and lentil [9]. Similarly, enzymatic hydrolysis of meat by-products has yielded peptides with multifunctional activities, including antioxidant, antihypertensive, and hypoglycemic effects [10]. Given their bioactivity and favorable functional properties, these peptides hold significant promise as ingredients in functional foods and nutraceuticals [11].

Compared to terrestrial mammals, gelatin derived from aquatic animals such as fish and amphibians offers distinct advantages, including greater resource availability, lower processing costs, and improved biosafety profiles [12,13]. *Lithobates catesbeianus* (American bullfrog), an economically important amphibian species native to North America, was introduced into Chinese aquaculture in the 1950s [14]. By 2024, China had become the world’s leading producer, accounting for over 90% of global output, with annual production reaching 800,000 tons. Owing to its aquatic habitat, the skin of *L. catesbeianus* displays remarkable biochemical diversity. Numerous studies have identified a variety of bioactive peptides in its skin secretions [15], typically composed of 8–48 amino acid residues, exhibiting diverse biological functions such as antimicrobial [16], anticancer [17], antiviral [18], immunomodulatory [19,20], antidiabetic [21,22,23], and antioxidant activities [24]. Interestingly, in addition to these secretions, *L. catesbeianus* skin is also a rich source of gelatin [25,26], with a unique amino acid profile containing high levels of Glu, Lys, Pro, and Arg, as reported by Ruiz Haddad et al. [27]. Previous research has demonstrated that peptides containing hydrophobic and basic amino acid residues are capable of forming hydrogen bonds and hydrophobic interactions with the active site of dipeptidyl peptidase-IV (DPP-IV), thus inhibiting its enzymatic activity [28]. However, despite the known bioactivity of *L. catesbeianus* skin peptides and the gelatin-rich nature of its skin, no studies to date have explored the DPP-IV inhibitory potential of gelatin hydrolysates derived from *L. catesbeianus* skin. This represents a significant knowledge gap in the current literature.

In this study, we prepared *L. catesbeianus* skin gelatin hydrolysates (LSGHs) using various proteases to release potential bioactive peptides. The peptide composition of the hydrolysates was characterized using LC-MS/MS. Subsequently, *in silico* screening was employed to identify novel DPP-IV inhibitory peptides. Selected candidates were synthesized and their inhibitory activities and interaction mechanisms with DPP-IV were further validated through biochemical assays. This work provided the first systematic investigation of DPP-IV inhibitory peptides derived from amphibian skin gelatin, offering new insights into the functional food potential of *L. catesbeianus*.

## 2. Materials and Methods

### 2.1. Materials

*L. catesbeiana* skin was purchased from a local aquatic market in Chaozhou, Guangdong, China. Bromelain and papain were obtained from Guangxi Pangbo Bioengineering Co., Ltd. (Nanning, Guangxi, China). Flavourzyme and Alcalase were purchased from Novozymes (Beijing, China), while alkaline protease, trypsin, and neutral protease were sourced from Beijing Solarbio Science & Technology Co., Ltd. (Beijing, China). Gly-Pro p-nitroanilide hydrochloride (G0513), Dipeptidyl Peptidase IV (D3446), Cytochrome c (C7752), Aprotinin (10820), Bacitracin (1951-M), Gly-Gly-Tyr-Arg (G5386), and Gly-Gly-Gly (G1377) were acquired from Sigma-Aldrich (Shanghai, China). IPI (S26288) was purchased from Shanghai Yuanye Biotechnology Co., Ltd. (Shanghai, China). Acetonitrile (ACN), trifluoroacetic acid (TFA), and formic acid (FA) were of high-performance liquid chromatography–mass spectrometry (HPLC-MS)-grade obtained from Merck (Darmstadt, Germany). Sephadex G25 was purchased from White Shark Biotechnology Co., Ltd. (Hefei, Anhui, China). All other reagents were of chromatographic or analytical grade. Peptides (purity > 95%) were synthesized by Synpeptide Co., Ltd. (Nanjing, Jiangsu, China).

### 2.2. Preparation of L. catesbeiana Skin Gelatin Hydrolysates (LSGHs)

The preparation of LSGHs was carried out following the method described by Xu et al. [29]. Frozen *L. catesbeiana* skin, previously stored at −20 °C, was thawed and gently washed with tap water to remove surface impurities. After draining, the skin was soaked in 0.05 M NaOH solution (1:5, *w*/*v*) at room temperature for 1.5 h to eliminate non-gelatin proteins and pigments. Subsequently, the skin was washed thoroughly with tap water until a neutral pH was reached, drained, and cut into small pieces. The pieces were then boiled in water (1:3.5, *w*/*v*) at 95 °C for 3 h to extract gelatin. The hot gelatin extract was filtered through two layers of 200-mesh cheesecloth to remove insoluble impurities. Finally, the filtrate was aliquoted and stored at −20 °C for subsequent use.

The *L. catesbeiana* skin gelatin was hydrolyzed using various proteases, including bromelain, Flavourzyme, Alcalase, alkaline protease, papain, trypsin, and neutral protease. A 100 g portion of gelatin solution (2.7%, *w*/*v*) was adjusted to the optimal pH for each enzyme, as listed in Table S1. Enzymic hydrolysis was performed by adding the respective enzyme at an enzyme-to-substrate ratio of 4.8% (*w*/*w*), and the reaction was carried out for 4 h at the optimal temperature for each enzyme (Table S1). Following hydrolysis, the gelatin solution was heated in boiling water for 10 min to inactivate the enzymes. The hydrolysate was then cooled to room temperature under running water and centrifuged at 8000 r/min for 10 min at 4 °C. The resulting supernatant was collected and freeze-dried. The obtained powder was stored at −20 °C for subsequent experiments.

### 2.3. Degree of Hydrolysis (DH) and Protein Recovery (PR)

The DH was determined using the o-phthaldialdehyde (OPA) method, as described by Wang et al. [30]. The extent of hydrolysis of *L. catesbeiana* skin gelatin by different proteases was quantified. Samples were diluted based on their protein content and the expected DH values. A serine solution (0.97 mM) was used as the standard for calibration.

The protein recovery rate was determined using the Kjeldahl method, as described by Zhu et al. [31]. The protein recovery after the hydrolysis of *L. catesbeiana* skin gelatin by different proteases was subsequently measured. The protein recovery rate was calculated using the following formula:

Protein recovery rate (%) = ω1 × 100/ω2

where ω1 refers to the protein content in the supernatant of the enzymatic hydrolysate, and ω2 refers to the protein content of *L. catesbeiana* skin used for the reaction.

### 2.4. DPP-IV Inhibition Assay

The inhibitory effect of LSGHs on DPP-IV enzyme activity was determined according to the method described by Zheng et al. [32]. Briefly, 80 μL of 0.5 mM p-nitroanilide substrate solution was mixed with 80 μL of peptide solution (final concentration: 0.8 mg/mL). The mixture was incubated at 37 °C for 10 min, followed by the addition of 40 μL of DPP-IV enzyme solution (0.8 U/μL). The absorbance at 405 nm was monitored every 2 min for 90 min. All reaction components were prepared in Tris-HCl buffer (100 mM, pH 8.0). A control reaction was prepared by replacing the peptide solution with Tris-HCl buffer. The experiment was repeated three times. The DPP-IV inhibition rate was calculated using the following formula:

DPP-IV inhibition rate (%) = (Slope of control group—Slope of sample group) × 100/Slope of control group.

Slope = (A2 − A1)/(T2 − T1).

where Slope represents the slope of the change in absorbance values A1 and A2 at two time points T1 and T2 within the linear range.

### 2.5. Molecular Weight Distribution

A peptide solution (10 mg/mL) was prepared, centrifuged, and then filtered through a 0.22 μm membrane for subsequent analysis. The molecular weight distribution of the peptides was analyzed using a HPLC system equipped with a 2487 UV detector and Empower workstation GPC software (2695, Waters Corp., Milford, MA, USA). Chromatographic separation was performed on a TSKgel 2000 SWXL column (300 mm × 7.8 mm). The mobile phase consisted of acetonitrile/water/trifluoroacetic acid (40:60:0.1, v/v/v). The detection wavelength was set at 220 nm, the flow rate was 0.5 mL/min, and the column temperature was maintained at 30 °C. Standard calibration curves were constructed using molecular weight markers including cytochrome c (MW 12,384), aprotinin (MW 6511), bacitracin (MW 1422), glycine-glycine-tyrosine-arginine (MW 451), and glycine-glycine-glycine (MW 189), all obtained from Sigma-Aldrich.

### 2.6. Size Exclusion Chromatography (SEC) Separation

The method of Zhou et al. [33] was followed with slight modifications. A solution of LSGHs hydrolyzed by papain (200 mg/mL) was prepared and filtered through a 0.45 μm membrane. A 5 mL aliquot of the solution was loaded onto a Sephadex G25 (2.5 cm × 60 cm, 5.0 μm). Elution was carried out at 4 °C using 0.01 M HCl as the mobile phase at a flow rate of 5 mL/17 min. The eluate was collected with a fraction collector, with 5 mL per tube. The absorbance of each fraction at 214, 254, and 280 nm was determined to generate a chromatogram profile. Fractions were pooled every five tubes to obtain a total of 33 combined fractions (F1–F33), which were then freeze-dried and stored at −20 °C for further analysis.

### 2.7. LC–MS/MS Analysis

After freeze-drying, the F8 sample was reconstituted to a final concentration of 25 μg/mL and filtered through a 0.22 μm membrane, and 2 μL was injected into an EASY-nLC 1200 nano LC system (Thermo Fisher Scientific Inc., Waltham, MA, USA). Sample enrichment was first conducted using an Acclaim Pep Map^TM^ 100 trap column (75 μm × 2 cm, nano Viper 2Pk C18, 3 μm, 100 Å pore size), followed by separation with an Acclaim Pep Map^TM^ RSLC analytical column (5 μm × 25 cm, nano Viper C18, 2 μm, 100 Å pore size). The mobile phase consisted of solvent A (0.1% formic acid in water, v/v) and solvent B (0.1% formic acid in 80% acetonitrile, v/v). The elution was performed at a flow rate of 300 nL/min using the following gradient: 0–2 min, 3–8% B; 2–48 min, 8–26% B; 48–53 min, 26–35% B; 53–57 min, 35–100% B; 57–60 min, 100% B.

Mass spectrometry was performed in positive data acquisition mode. Full MS1 scans were acquired at a resolution of 70,000 FWHM across an m/z range of 100 to 1500. The top 20 most intense precursor ions were selected for MS2 analysis using high-energy collision dissociation (HCD) with stepped normalized collision energies of 10%, 25%, and 45%. Fragment ions were detected at a resolution of 17,500 FWHM using a quadrupole isolation window of 1.6 m/z.

Raw data were analyzed using the open-source search engine pFind 3.2.1 software (Beijing, China) [34]. The peptide mass tolerance was set at ±10 ppm, and the fragment mass tolerance at ±0.05 Da. Peptides with molecular weights of 200–10,000 Da and chain lengths of 2–100 amino acids were included in the screening.

### 2.8. In Silico Analysis and Screening of Potential DPP-IV Inhibitory Peptides

The bioactivity, cell permeability, similarity to known bioactive peptides, hydrophilicity and predicted half-life of the screened peptide sequences were evaluated using the following tools: PeptideRanker (http://distilldeep.ucd.ie/PeptideRanker/, accessed on 15 October 2024); a threshold of 0.5 was used, where values above 0.5 indicate superior properties and those below 0.5 suggest inferior ones), CPPpred (http://distilldeep.ucd.ie/CPPpred/, accessed on 15 October 2024); with a threshold of 0.5, values > 0.5 denote better performance and values < 0.5 imply worse performance), the BIOPEP-UWM database (https://biochemia.uwm.edu.pl/biopep-uwm/, accessed on 15 October 2024); if no hits were retrieved, the peptide was considered novel), and ADMETlab 3.0 (https://admetlab3.scbdd.com/, accessed on 16 October 2024); a solubility (logS) between −4 and 0.5 was regarded as moderate; logS < −4 indicates poor solubility, while logS > 0.5 suggests excessively high solubility). The peptide sequences were then converted into three-dimensional structures using ChemDraw 20.0 software (PerkinElmer Inc., Cambridge, MA, USA). The crystal structure of DPP-IV (entry code: 1WCY) was preprocessed using PyMol software (Open source version) (DeLano Scientific LLC, San Francisco, CA, USA). Molecular docking between the peptides and DPP-IV was subsequently carried out using the Smina software package. The docking grid was centered at the active site of DPP-IV (coordinates: x = 48.69, y = 59.87, z = 31.85), with a box size of 71.75 × 69.47 × 71.75 and a grid spacing of 0.375 Å. The exhaustiveness parament was set to 24 to obtain the binding affinity between the peptide sequences and the protein. The docking results were analyzed in PyMol software to assess the binding ability and type between the peptide sequences and the protein.

Molecular dynamics (MD) simulations of the peptide–DPP-IV complexes were conducted using GROMACS version 2020.6, following the protein–ligand complex tutorial available on the GROMACS Tutorials website (http://www.mdtutorials.com/gmx/index.html, accessed on 22 October 2024) [35]. The simulation protocol included standard preprocessing steps: generating of topology files, definition of the docking box and solvent, adding ions, energy minimization, and equilibration of temperature and pressure. The production MD run was performed with a time step of 2 fs for a total of 50,000,000 steps, corresponding to a simulation time of 100 ns. Trajectory data were collected throughout the simulation for further analysis [36].

### 2.9. Validation of Peptide Sequence Synthesis

The peptide sequences LGPQR, RGFDQ, RGPVGP, and RLDDVT were synthesized by Synpeptide Co., Ltd. (Nanjing, China) using the solid-phase peptide synthesis method. To investigate the inhibition mechanisms of these peptides against DPP-IV, enzyme kinetics experiments were performed. Lineweaver–Burk double reciprocal plots were generated by plotting the reciprocal of substrate concentrations (1/[S]) against the reciprocal of reaction rates (1/V). The kinetic parameters, including *v_max_*, *K_m_*, *K_i_*, and *K*_is_, were calculated to determine the modes of inhibition [37,38].

### 2.10. Statistical Analysis

Statistical analysis was performed using SPSS 22.0 software package (SPSS Inc., Chicago, IL, USA). The results are presented as mean ± standard error of the mean (SEM). Duncan’s multiple comparison test was used for significance analysis and *p* < 0.05 was considered statistically significant.

## 3. Results and Discussion

### 3.1. Physicochemical Property of Different LSGHs

Papain and bromelain are both classified as thiol endopeptidases. Papain preferentially cleaves peptide bonds where a bulky hydrophobic amino acid occupies the P2 position (EC 3.4.22.2), whereas bromelain exhibits broad substrate specificity with no strong preference for particular residues (EC 3.4.22.33). Among the seven proteases tested, Flavourzyme was the only one possessing both endopeptidase and exopeptidase activities. Its exopeptidase function is attributed to Leu aminopeptidase, which specifically hydrolyzes peptide bonds formed by N-terminal Leu residues [39]. Alcalase, subtilisin, and Neutrase were all extracellular bacterial proteases isolated from *Bacillus subtilis*, yet they exhibit distinct substrate preferences. Alcalase and subtilisin (EC 3.4.21.62) preferentially hydrolyzed peptide bonds involving neutral amino acid at the P1 position [40], whereas Neutrase preferentially targets peptide bonds of Xaa-|-Leu and Xaa-|-Phe (EC 3.4.24.27). Trypsin, the only animal-derived protease in the group, preferentially cleaves peptide bonds at the carboxyl side of arginine (Arg-|-Xaa) and lysine (Lys-|-Xaa) residues (EC 3.4.21.4).

**Figure 1:**
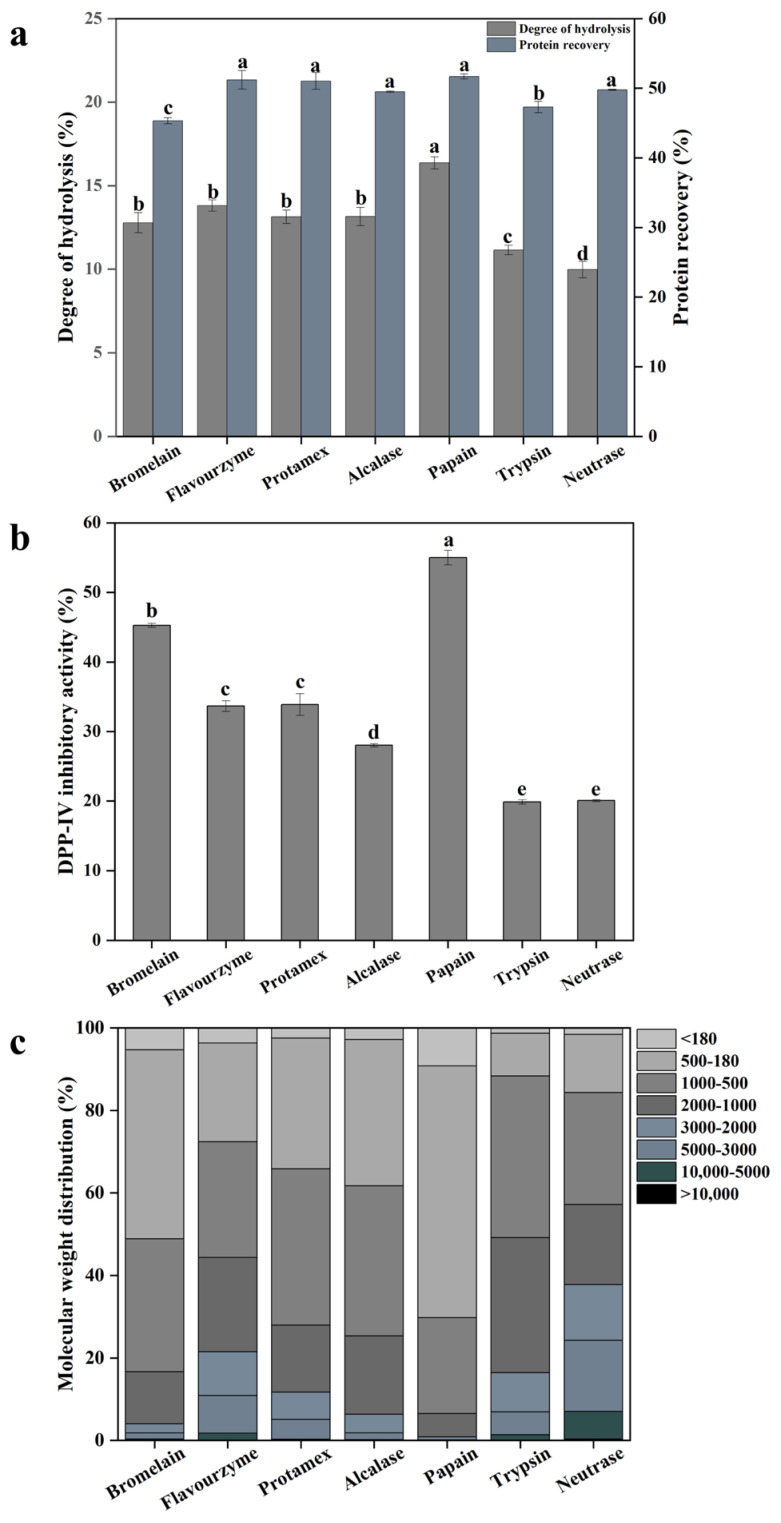
Degree of hydrolysis (DH) and protein recovery (PR) (**a**), dipeptidyl-peptidase IV (DPP-IV) inhibition rate (**b**), and molecular weight distribution (**c**) of LSGHs. Different LSGHs were named after enzymes employed. Bars represent standard deviations from triplicate determination. Different letters above the bars indicate significant differences at *p* < 0.05.

As shown in Figure 1a, significant differences (*p* < 0.05) were observed in the degree of hydrolysis (DH) among the LSGHs obtained from different proteases, with values ranging from 9.98% to 16.36%. Papain exhibited the highest hydrolytic efficiency (16.36 ± 0.37%), which was 63.93% higher than that of Neutrase, the enzyme with the lowest DH. In addition, the protein recovery (PR) from the raw material ranged from 45.32% to 51.67%. No significant difference (*p* > 0.05) in PR values were observed among the groups hydrolyzed by Flavourzyme, Alcalase, subtilisin, papain, and Neutrase (all approximately 49% to 51%). The highest PR was obtained from the papain-hydrolyzed group, which was 14.01% higher than that of the bromelain-hydrolyzed group, which showed the lowest PR. The relatedly narrow range of DH and PR values observed across all proteases might be attributed to substrate depletion during the extended hydrolysis process, which likely led to system saturation and limited further enzymatic activity.

### 3.2. DPP-IV Inhibitory Activity of LSGHs

As shown in Figure 1b, when the final protein concentration of LSGHs was 0.8 mg/mL, the highest DPP-IV inhibition rate was observed in the papain-hydrolyzed group (54.98 ± 1.04%), followed by the bromelain-hydrolyzed group (45.26 ± 0.30%). The LSGHs prepared using Flavourzyme, Alcalase, and subtilisin showed moderate inhibition, ranging from 28% to 34%. In contrast, the lowest inhibitory activities were recorded for LSGHs hydrolyzed by trypsin and Neutrase, both with inhibition rates around 20%. There were significant differences (*p* < 0.05) in DPP-IV inhibitory activity among the seven groups, with a maximum variation of 34.98%. These results were consistent with those reported by Xu et al. [29], who found that papain hydrolysis produced peptides with the strongest DPP-IV inhibitory activity, whereas peptides generated by neutral protease hydrolysis exhibited the weakest effect in a study on tilapia skin gelatin. Interestingly, in this study, a comparable DPP-IV inhibitory effect to that reported by Xu et al. [29] at 1 mg/mL was achieved using only 0.8 mg/mL of LSGHs, suggesting that LSGHs may possess a stronger inhibitory potential against DPP-IV activity.

### 3.3. Molecular Weight Distribution of LSGHs

Previous studies have demonstrated that peptides with molecular weights below 1 kDa exhibited the highest DPP-IV inhibitory activity [41]. As shown in Figure 1c, the papain-hydrolyzed group had the highest proportion of peptides under 1 kDa (93.47%), followed by the bromelain group (83.34%). These results are consistent with those in Section 3.2, in which the papain group showed the strongest DPP-IV inhibitory activity, with bromelain ranking second. Similarly, Li-Chan et al. [42] reported that, at equivalent concentration, the <1 kDa fraction exhibited significantly greater DPP-IV inhibition compared to the 1–2.5 kDa and >2.5 kDa fractions (*p* < 0.05).

However, the results in Section 3.2 also showed that the DPP-IV inhibitory activity of the Flavourzyme group was comparable to that of the Alcalase group and even higher than that of the subtilisin group. In contrast, the molecular weight distribution revealed that both the Alcalase (72.07%) and subtilisin (74.63%) groups contained higher proportions of peptides under 1 kDa than the Flavourzyme group (55.67%). A similar inconsistency was observed by Xu et al. [29] in their study on TSGHs. As noted by Wang et al. [1], peptide molecular weight alone was not a sufficient predictor of DPP-IV inhibitory activity. The amino acid sequence and three-dimensional structure of the peptides, along with their specific interactions with the enzyme, must also be taken into account.

### 3.4. Separation of DPP-IV Inhibitory Peptides Using SEC

**Figure 2:**
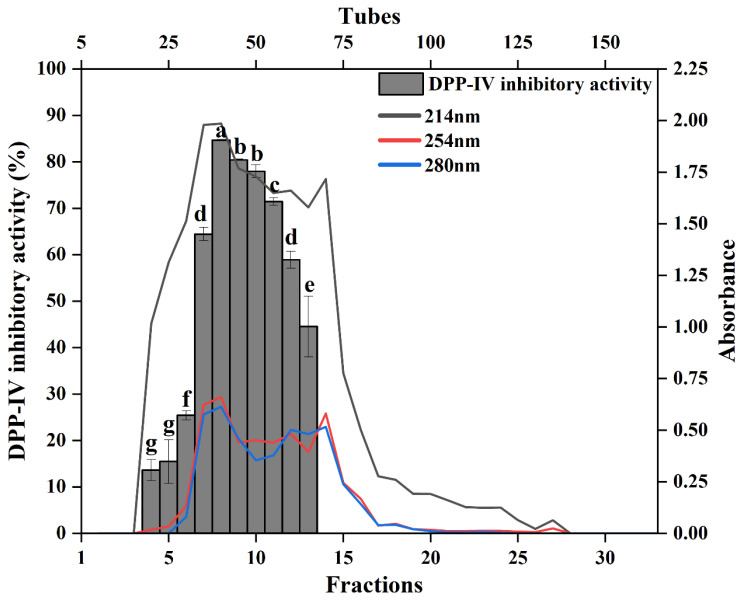
SEC chromatogram and DPP-IV inhibitory activity of different fractions. Bars represent standard deviations from triplicate determination. Different letters above the bars indicate significant differences at *p* < 0.05.

Size exclusion chromatography (SEC) is a technique that utilizes the porous structure of cross-linked dextran gels to separate components in a mixture based on molecular weight differences [43]. As shown in Figure 2, LSGHs were derived from the papain hydrolysate subjected to SEC using a Sephadex G25 column, resulting in the collection of 33 fractions by combining every five tubes into a single fraction. In this separation process, higher molecular weight components were eluted earlier, whereas lower molecular weight components were eluted later. UV detection was employed to monitor peptide content in each fraction, with fractions F4 to F20 exhibiting absorbance values greater than 0.2 at 214 nm (indicative of peptide bonds), 254 nm (associated with hydrophobic amino acids), and 280 nm (representing aromatic amino acids). Among these, fractions F4 to F13 demonstrated DPP-IV inhibitory activity. When evaluated at a final concentration of 0.8 mg/mL, fractions F8, F9, and F10 exhibited notably high DPP-IV inhibition rates of 84.67 ± 0.12%, 80.44 ± 0.17%, and 77.95 ± 1.40%, respectively. Remarkably, the DPP-IV inhibition rate of fraction F8 was 53.95% higher than that of the crude papain hydrolysate prior to Sephadex G25 separation. This result indicated that SEC using Sephadex G25 effectively enriched peptides with potent DPP-IV inhibitory activity.

### 3.5. Identification of DPP-IV Inhibitory Peptides

The raw data generated from EASY-nLC 1200 nano LC system were analyzed using pFind software [34] for peptide sequence identification. In the F8 fraction described in Section 3.4, 22 unique peptide sequences were identified. Following computational screening, 13 candidate peptide sequences were selected, as summarized in Table 1. Peptides had molecular weights ranging from 525 to 768 Da and consisted of 5 to 8 amino acid residues. This suggests that the DPP-IV inhibitory peptides in the F8 fraction predominantly consisted of short peptides (5–8 peptides) with molecular weights below 1 kDa, which was consistent with the molecular weight distribution observed in the papain hydrolysate group described in Section 3.3.

The predicted biological scores of the 13 peptides ranged from 0.11 to 0.69, and their predicted cell permeability scores ranged from 0.11 to 0.66. Interestingly, a negative correlation was observed in some cases, where peptides with higher bioactivity scores exhibited lower cell permeability, and vice versa. This phenomenon has also been reported by Wang et al. [30] in a study involving computer-assisted screening of casein-derived peptides. Further ADMET profiling of the 13 peptides using the ADMETlab 3.0 platform revealed that all peptides exhibited favorable water solubility (−4 < logS < 0.5) and short predicated half-lives (T_1/2_), indicating their potential for rapid absorption and metabolism when administered orally.

Molecular docking simulations were performed using the Smina software (based on (Autodock Vina) to evaluate the binding affinity of each peptide with DPP-IV (1WCY). The resulting binding energies ranged from −9.1 to −6.8 kcal/mol. Binding energies below 0 kcal/mol suggest spontaneous interactions, and values below −5 kcal/mol are indicative of good binding affinity, supporting the potential inhibitory activity of these peptides. Based on pFind software [34] and the Uniprot database annotations, the precursor proteins of 13 identified peptides were primarily derived from three amphibian species: *Lithobates catesbeianus* (American bullfrog), *Xenopus laevis* (African clawed frog), and *Xenopus tropicalis* (Western clawed frog). The most frequently identified precursor protein was O42350 from the *L. catesbeianus*, which encodes the α2 chain of type I collagen.

### 3.6. Mechanism of Peptide–DPP-IV Interaction

**Figure 3:**
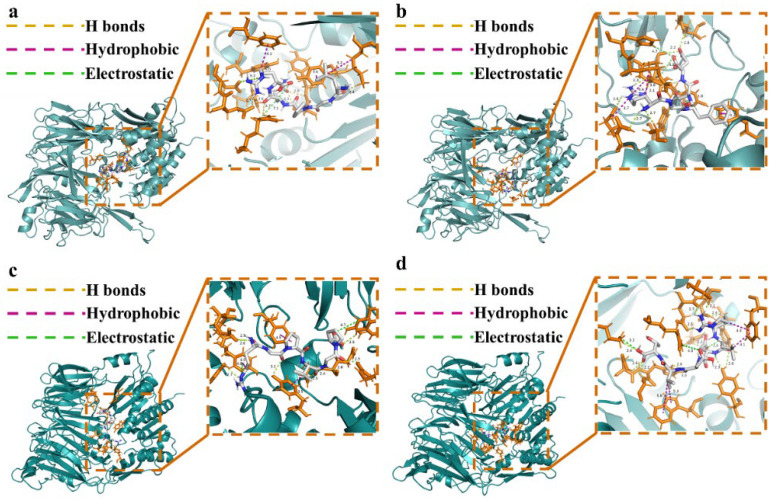
Molecular docking results of four inhibitory peptides with DPP-IV. (**a**) LGPQR; (**b**) RGFDQ; (**c**) RGPVGP; (**d**) RLDDVT. Yellow dashed lines are hydrogen bonds, purple dashed lines are hydrophobic interactions, green dashed lines are electrostatic interactions, and the numbers on the dashed lines are bond lengths.

Four DPP-IV inhibitory peptides with low binding energies derived from LSGHs were selected for detailed molecular docking analysis with DPP-IV, as illustrated in Figure 3 and summarized in Table 2. All four peptides were found to interact with the active site of DPP-IV through a combination of hydrogen bonding, hydrophobic interactions, and electrostatic forces. Notably, the N-terminal Leu residue in the peptide LGPQR has been previously reported to exhibit strong hydrophobic characteristics when positioned at the N-terminus, thereby facilitating hydrophobic interactions with DPP-IV [44]. In addition, the C-terminal Arg in LGPQR, as well as the N-terminal Arg residues in RGFDQ, RGPVGP, and RLDDVT, are basic hydrophilic amino acids, contributing to electrostatic and hydrogen bond interactions within the binding pocket. These findings are consistent with previous reports by Nongonierma et al. [2] for the APG peptide, and by Huang et al. [45] for the CAYQWQRPVDRIR peptide, both of which demonstrated similar interaction patterns with DPP-IV.

Previous studies have shown that DPP-IV possesses three key active pockets: S1 (Tyr547, Ser630, Tyr631, Val656, Trp659, Tyr662, Tyr666, Asn710, Val711, and His740), S2 (Glu205, Glu206, and Tyr662), and S3 (Ser209, Phe357, and Arg358) [46,47]. As depicted in Figure 3, all four peptides successfully docked into the active sites of DPP-IV. Specifically, LGPQR formed hydrogen bonds with hydrophilic residues Glu205, Ser630, Tyr631, and Tyr662, participated in Pi-Alkyl interactions with hydrophobic aromatic residues such as Phe357, Tyr547, and Tyr666, and engaged in electrostatic interactions with Glu206. RGFDQ formed hydrogen bonds with Glu205, Glu206, Arg358, and Tyr662, Pi-Alkyl interactions with Tyr547, and electrostatic interactions with Glu205 and Tyr666. RGPVGP formed hydrogen bonds with Glu205, Glu206, Tyr547, and Tyr662, Pi-Alkyl interactions with Phe357, Tyr547, and Tyr666, and electrostatic interactions with Tyr666. RLDDVT formed hydrogen bonds with Glu205, Glu206, Tyr547, Tyr662, and His740, Pi-Alkyl interactions with Phe357, and electrostatic interactions with Tyr666. These results suggested that Glu205, Glu206, Phe357, Tyr547, and Tyr662 are key residues mediating peptide–DPP-IV binding, predominantly through hydrogen bonding and hydrophobic interactions. To further evaluate the stability of these interactions, molecular dynamics simulations were subsequently performed for all four DPP-IV inhibitory peptides.

### 3.7. Stability Analysis of DPP-IV Inhibitory Peptide Binding to DPP-IV

**Figure 4:**
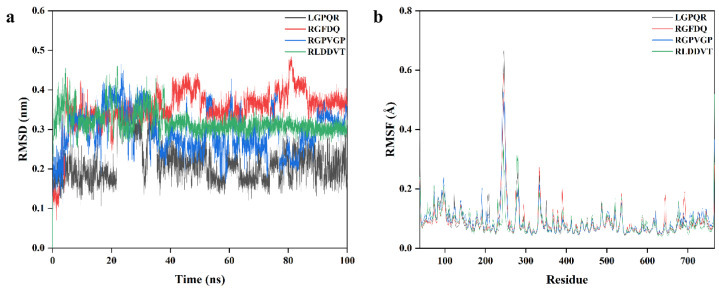
Molecular dynamics simulations of four inhibitory peptides with DPP-IV enzyme at 100 ns. (**a**) Root mean square deviation (RMSD) curves; (**b**) root mean square fluctuation (RMSF) curves.

The molecular dynamics simulation results of four DPP-IV inhibitory peptides are presented in Figure 4. Root mean square deviation (RMSD), a key metric for assessing the conformational stability of protein–ligand complexes, was employed to evaluate the dynamic stability of the peptide–DPP-IV systems. Over the 100 ns simulation period, the RMSD trajectories of the LGPQR, RGFDQ, RGPVGP, and RLDDVT complexes stabilized at approximately 50 ns, 38 ns, 40 ns, and 42 ns, respectively. After stabilization, all complexes maintained fluctuation within 0.2 nm (2 Å), indicating that each peptide formed a structurally stable complex with DPP-IV and did not undergo major conformational shifts. These results suggested that all four peptides exhibited good binding stability with DPP-IV, supporting their potential as effective DPP-IV inhibitors. Similar findings were reported by Liang et al. [48], who observed comparable RMSD behavior in complexes involving three different DPP-IV inhibitory peptides.

The flexibility of individual DPP-IV amino acid residues during the molecular dynamics simulations was evaluated using root mean square fluctuation (RMSF) analysis [49]. In all four peptide–DPP-IV complexes, residues 228–262, 270–288, 326–346, and 527–548 exhibited higher RMSF values compared to other regions, indicating greater structural flexibility in these segments during the simulation. Notably, the RMSF values of all residues in the four complexes were below 0.8 Å, suggesting that the overall conformational fluctuations were minimal and that the peptide–DPP-IV complexes remained structurally stable through the 100 ns simulation. These findings are consistent with those reported by Chen et al. [50], who observed similar RMSF patterns in their molecular dynamics analysis of shiitake-derived peptides as ACE and DPP-IV inhibitors.

### 3.8. Synthesis of Peptides and Confirmation of DPP-IV Inhibitory Activity

**Figure 5:**
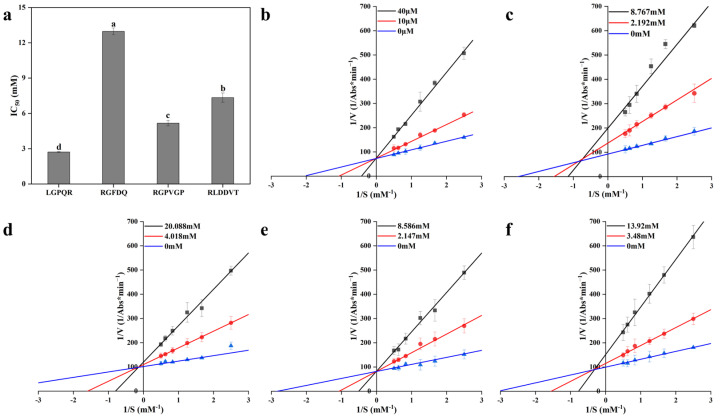
Synthesis of four inhibitory peptides to inhibit DPP-IV for validation analysis. (**a**) IC_50_ of four inhibitory peptides to inhibit DPP-IV; (**b**–**f**) Lineweaver–Burk plots of DPP-IV inhibition by IPI, LGPQR, RGFDQ, RGPVGP, and RLDDVT, respectively. Different letters above the bars indicate significant differences at *p* < 0.05.

**Figure 6:**
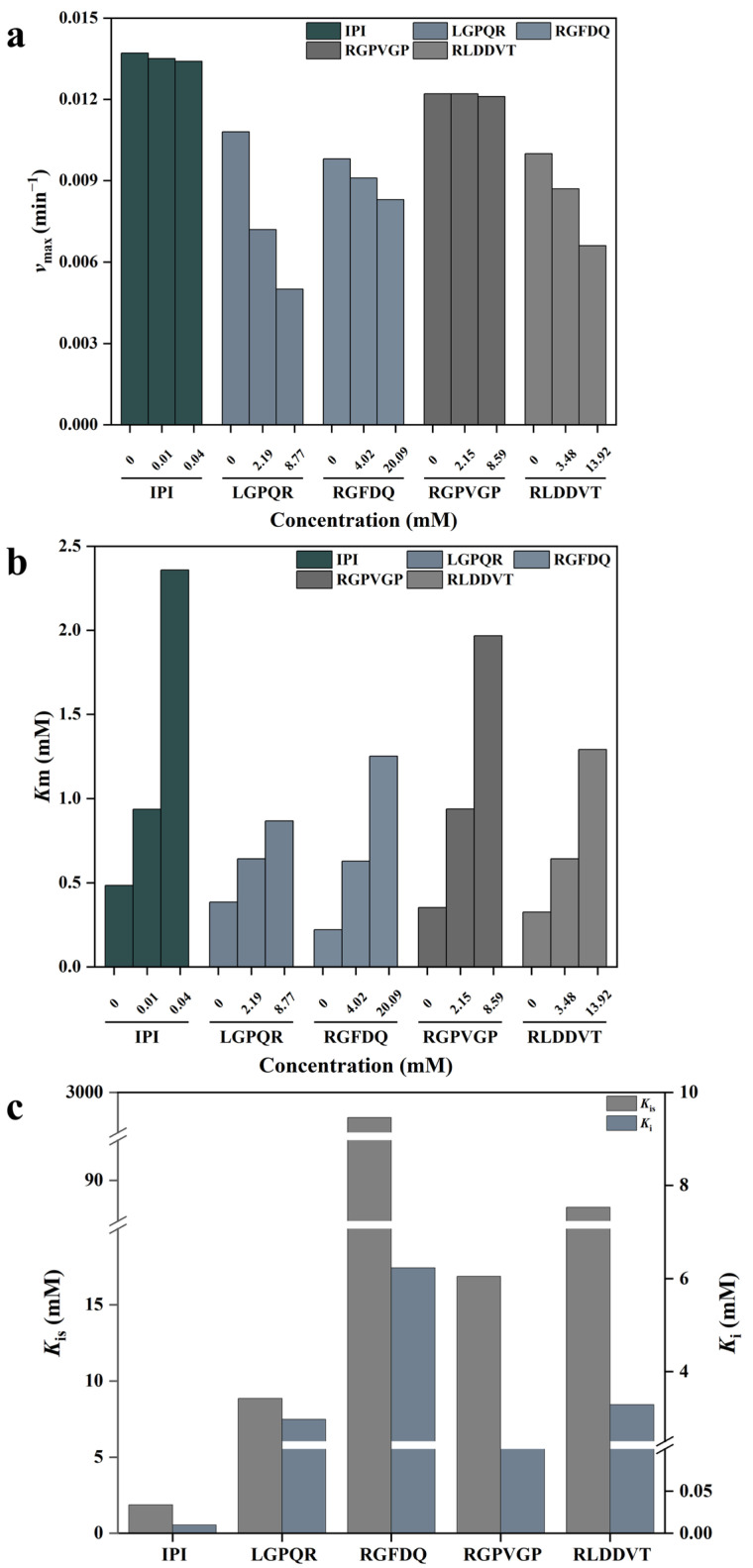
Kinetic parameters of DPP-IV inhibition by four inhibitory peptides: (**a**) maximum speed (*v_max_*); (**b**) Michaelis constant (*Km*); (**c**) inhibition equilibrium constants for inhibitor binding to free enzymes (*Ki*) and inhibitor binding to enzyme–substrate complexes (*Kis*), respectively.

To further confirm the DPP-IV inhibitory potential of the identified peptide sequences, four peptides were synthesized via solid-phase peptide synthesis, with IPI as a positive control. Their inhibitory activities and corresponding enzyme kinetics were evaluated, as presented in Figure S1, Figure 5, and Figure 6. The IC_50_ of IPI was determined to be 15.89 μM, consistent with previously reported values ranging from 3.5 to 210 μM [32,51,52]. The IC_50_ values of the four test peptides ranged from 2.72 to 12.97 mM, with inhibitory efficacy in the following order: LGPQR > RGPVGP > RLDDVT > RGFDQ. Notably, the IC_50_ of LGPQR was 28.98% lower than that of a peptide isolated from buffalo colostrum (3.83 mM) [53] and 44.03% lower than the most potent peptide derived from egg proteins (4.86 mM) [47]. This enhanced inhibitory activity may be attribute to the unique composition of LGPQR, which contains hydrophobic residues (Leu, Gly and Pro) and a basic residue (Arg), potentially promoting stronger binding interactions with DPP-IV [28].

To elucidate the mechanism by which inhibitory peptides act on DPP-IV, Lineweaver–Burk double reciprocal plots were employed to determine the nature of enzyme–inhibitor interaction [3]. As shown in Figure 5b and Figure 6a,b, the Lineweaver–Burk plots of IPI with DPP-IV exhibited a common intersection on the positive *Y*-axis. In this case, the *v_max_* remained unchanged while the *K_m_* increased with increasing concentration of IPI, indicating a typical competitive inhibition mechanism [54]. Similarly, Figure 5e shows that RGPVGP also exhibited competitive inhibition, as its Lineweaver–Burk plot intersected the positive *Y*-axis, and the variations in *v_max_* and *K_m_* were consistent with those observed for IPI. In contrast, Figure 5c,d,f depict the double reciprocal plots for LGPQR, RGFDQ, and RLDDVT, respectively, which intersected in the second quadrant (between the positive *Y*-axis and the negative *X*-axis). These plots revealed that *v_max_* increased while *K_m_* decreased with rising inhibitor concentrations, suggesting a mixed-type inhibition model that involves both competitive and non-competitive binding modes [54]. A similar finding was reported by Liu et al. [55] in their investigation of DPP-IV inhibition by tilapia skin collagen peptides, where it was proposed that peptides could bind both to the enzyme’s active site and to allosteric sites to exert inhibitory effects [56]. Furthermore, *K_i_* and *K_is_* represent the inhibition constants for inhibitor binding to the free enzyme and the enzyme–substrate complex, respectively [57]. As shown in Figure 6c, both IPI and the four synthesized peptides exhibited *K_is_* > *K_i_*, indicating that these peptides had a higher affinity for the free enzyme than for the enzyme–substrate complex [58].

## 4. Conclusions

Gelatin extracted from *L. catesbeiana* (American bullfrog) skin via high-temperature hot water treatment demonstrated strong potential as a precursor for DPP-IV inhibitory peptides. Through efficient enzymatic hydrolysis, hydrolysates with a high degree of hydrolysis, elevated protein recovery rate, and notable DPP-IV inhibitory activity were obtained. Following purification using SEC, 13 bioactive peptides with favorable cell permeability were identified by nano LC-MS/MS. All 13 peptides exhibited spontaneous and stable binding to the active sites of DPP-IV, primarily mediated by hydrogen bonding and hydrophobic interactions. Among them, four peptides, namely LGPQR, RGFDQ, RGPVGP, and RLDDVT, demonstrated the strongest binding affinities. Subsequent synthetic validation confirmed that these peptides functioned as competitive or mixed-type (competitive/non-competitive) inhibitors of DPP-IV. Despite these promising results, the *in vivo* mechanisms through which LSGHs influence glucose metabolism and the associated regulatory pathways remain unclear and warrant further investigation.

## Figures and Tables

**Table 1:** Peptides sequence identification and activity prediction of *L. catesbeiana* skin gelatin hydrolysates (LSGHs).

Peptide Sequence	Mw (Da)	RT (min)	Peptide Ranker Score ^a^	CPPpred Score ^b^	logS ^c^	T_1_/_2_^d^	Affinity (kcal/mol)	Accessions ^e^
INTPVK	671.41	9.37	0.12	0.22	−1.33	1.16	−7.4	P15438
KNLVLT	687.44	11.52	0.12	0.66	−1.26	1.09	−6.8	Q28GH3, Q642Q1, Q7ZY60
LGPDGR	614.33	10.8	0.55	0.24	−2.32	1.02	−7.5	Q8AVH7
LGPQR	570.34	9.37	0.52	0.55	−1.98	0.81	−8.2	Q498F0
LPGPDGR	727.37	9.92	0.65	0.21	−2.62	1.04	−7.8	O42350
MGPVGPR	729.37	9.42	0.69	0.31	−2.37	0.94	−8.0	O42350
PVGPR	525.31	10.59	0.55	0.44	−2.09	1.13	−7.5	O42350
RGEGLPA	699.38	10.03	0.30	0.32	−2.03	1.02	−7.2	Q58HI1
RGFDQ	622.30	10.48	0.47	0.11	−3.05	1.04	−9.1	Q6P1W0
RGPVGP	582.34	9.65	0.51	0.35	−1.92	0.82	−8.7	O42350
RLDDVT	718.37	15.69	0.11	0.30	−2.06	1.53	−8.4	Q5U538
SVGPVGPR	768.44	13.96	0.50	0.23	−2.04	1.17	−7.3	O42350
VGPVGPR	681.4	11.3	0.52	0.38	−2.07	0.87	−8.0	O42350

^a^ from PeptideRanker (http://distilldeep.ucd.ie/PeptideRanker/, accessed on 15 October 2024). ^b^ from CPPpred (http://distilldeep.ucd.ie/CPPpred/, accessed on 15 October 2024). ^c,d^ from ADMETlab 3.0 (https://admetlab3.scbdd.com/, accessed on 16 October 2024). ^e^ from Uniprot (https://www.uniprot.org/, accessed on 16 October 2024).

**Table 2:** Peptide sequence of *L. catesbeiana* skin collagen with DPP-IV binding site.

	Hydrogen Bonds	Hydrophobic	Electrostatic
LGPQR	Arg125, Glu205, Lys554, Ser630, Tyr631, Tyr662	Phe357, Tyr547, Lys554, Tyr666	Arg125, Glu206
RGFDQ	Glu205, Glu206, Arg358, Tyr662, Asp709, Asp739, Gly741	Arg125, Tyr547	Arg125, His126, Glu205, Tyr666, Asp709
RGPVGP	Glu205, Glu206, Tyr456, Ser552, Gln553, Tyr547, Arg560, Tyr662	Phe357, Tyr547, Tyr666	Arg429, Tyr666
RLDDVT	Lys122, Glu205, Glu206, Ser209, Tyr547, Tyr662, Asp739, His740, Gly741	Phe357, Trp629	Lys122, Arg125, Tyr666

## Data Availability

The original contributions presented in this study are included in the article/Supplementary Materials. Further inquiries can be directed to the corresponding author.

## Supplementary Materials

The following supporting information can be downloaded at: https://www.mdpi.com/article/10.3390/foods14173023/s1, Figure S1: Graph of the inhibitory effect of IPI on DPP-IV enzyme; Table S1: Optimum pH and temperature for the enzymes employed.
