# Supplementary material for: Uncovering Novel DPP-IV Inhibitory Peptides from Amphibian (Lithobates catesbeiana) Skin via Peptidomics and Molecular Simulation
Source: Foods. 2025 Aug 28;14(17):3023. doi: 10.3390/foods14173023 (PMC12427844; doi:10.3390/foods14173023)
Supplement: Supplementary file 1 [file foods-14-03023-s001.zip › foods-3824089-supplementary.pdf]

Supplementary Materials

Uncovering Novel DPP-IV Inhibitory Peptides from Amphibian (*Lithobates catesbeiana*) Skin via Peptidomics and Molecular Simulation

Zongmu Fang[1], Mei Zhang [1,*], Junhui Lian [1], Yangqing Xiao [1], Donghui Luo [1], Mouming Zhao [1,2], Lianzhu Lin [1,2,]**

1 Chaozhou Branch of Chemistry and Chemical Engineering Guangdong Laboratory, Chaozhou, Guangdong, China

2 School of Food Science and Engineering, South China University of Technology, Guangzhou, 510640, China

* Corresponding author. Chaozhou Branch of Chemistry and Chemical Engineering Guangdong Laboratory, Chaozhou, 521000, China.

** Corresponding author. School of Food Science and Engineering, South China University of Technology, Guangzhou, 510640, China.

Table S1
Optimum pH and temperature for the enzymes employed.

Enzymes	pH	Temperature (℃)
Bromelain	6.8	45
Flavourzyme	7.5	50
Protamex	7.5	50
Alcalase	10.5	45
Papain	6.5	55
Trypsin	7.5	37
Neutrase	7.0	50

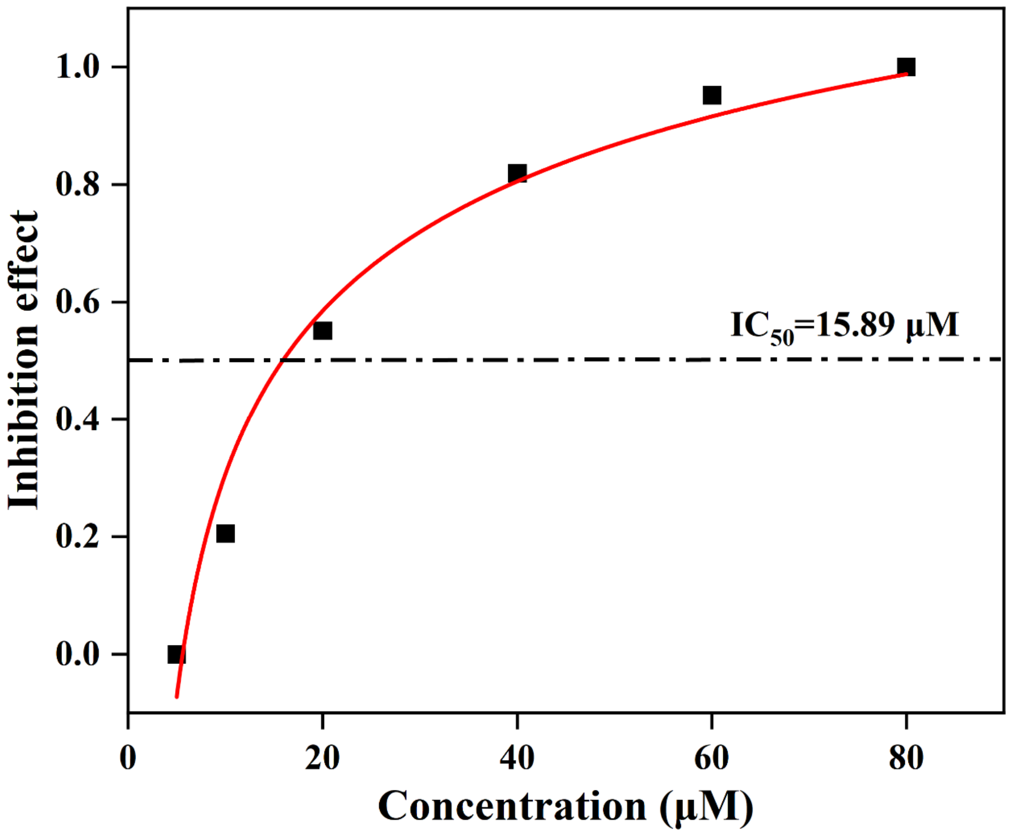

Figure S1. Graph of the inhibitory effect of IPI on DPP-IV enzyme
